# Longitudinal Genotype Replacement, Recombination and Neutralizing Epitope Variations in Porcine Epidemic Diarrhea Virus at a Commercial Pig Farm in Guangxi, China

**DOI:** 10.3390/ani16203262

**Published:** 2026-10-16

**Authors:** Feng Qin, Yang Luo, Jianzhu Li, Zhili Li, Jianguo Dong, Zhehui Qu, Guangke Xu, Tao Liu, Kaiwei Deng, Chihai Ji

**Affiliations:** 1 College of Animal Science and Technology, Xinyang Agriculture and Forestry University, Xinyang 464000, China; 2024200001@xyafu.edu.cn (F.Q.); lijianzhu78@163.com (J.L.); 2019200001@xyafu.edu.cn (Z.L.); dongjianguo213@163.com (J.D.); quzhehui1980@xyafu.edu.cn (Z.Q.); xuguangke2005@126.com (G.X.); decai178@163.com (T.L.); dkwyjs@163.com (K.D.); 2 Research Institute of Wens Foodstuff Group Co., Ltd., Yunfu 527400, China; 402710820@139.com; 3 South China Sea Fisheries Research Institute, Chinese Academy of Fishery Sciences, Guangzhou 510300, China

**Keywords:** porcine epidemic diarrhea virus, spike gene, homologous recombination, neutralizing epitope, N-glycosylation, antigenic drift, longitudinal surveillance

## Abstract

Porcine epidemic diarrhea virus (PEDV) causes severe enteric disease in piglets. The spike (S) gene is the major target of neutralizing antibodies and a hotspot for genetic variation. In this study, eight PEDV S-gene sequences were obtained from a commercial pig farm in Guangxi, China, between 2021 and 2025. Phylogenetic analysis assigned all sequences to the G2 genotype, with G2c, G2d, and G2b subclusters detected at different time points. Recombination analysis identified sequence-level recombination signatures in the G2c sequences, with G2b-like and G2d-like sequences as parental relatives. Several amino acid substitutions were located within or adjacent to the COE and SS2 neutralizing epitopes, and N-glycosylation site variations were observed among subclusters. Structural predictions suggest that these combined changes may alter surface antigenic properties and confer immune evasion potential, though functional validation using neutralization assays is still required. These findings provide field-derived evidence for PEDV genetic evolution and recombination within a single swine herd, and support rational vaccine-strain evaluation and on-farm biosecurity strategies.

## Simple Summary

Porcine epidemic diarrhoea virus (PEDV) causes devastating watery diarrhoea in neonatal piglets and results in substantial economic losses to the swine industry. Long-term sow vaccination may exert persistent immune pressure that potentially contributes to viral evolution and inter-lineage homologous recombination. In this five-year longitudinal investigation, eight PEDV field isolates were recovered from a single closed commercial pig farm in Guangxi, China. Phylogenetic and recombination analyses demonstrated obvious temporal genotype turnover within the herd: endemic G2d variants prevailed during 2021–2022, followed by the emergence and predominance of novel recombinant G2c strains after 2023. All G2c isolates arose from natural homologous recombination between indigenous farm G2d strains and externally introduced G2b isolates. Fixed amino acid substitutions located in key neutralizing epitopes, combined with systematic N-glycosylation remodelling, reshaped the surface antigen profiles of PEDV, which suggests potential humoral immune evasion capacity. This long-term field investigation depicts a temporal evolution of farm-resident PEDV and provides field-based evidence to support rational vaccine strain updating and targeted on-farm prevention against porcine epidemic diarrhoea.

## 1. Introduction

Porcine epidemic diarrhoea (PED) is an acute, highly contagious intestinal disease caused by porcine epidemic diarrhoea virus (PEDV). Infected neonatal piglets develop severe watery diarrhoea, vomiting and lethal dehydration; mortality can reach 80–100% for piglets infected within seven days after birth [1,2]. Since highly virulent PEDV variants emerged in 2010, repeated outbreaks have swept major pig-raising regions across Asia and North America, leading to substantial economic losses for the global swine industry [3,4].

PEDV belongs to the genus *Alphacoronavirus*. It possesses a positive-sense single-stranded RNA genome of approximately 28 kb, which encodes four structural proteins (S, E, M, N) together with the auxiliary ORF3 protein [5]. The spike (S) glycoprotein mediates host-receptor binding and membrane fusion and represents the primary target for host neutralizing antibodies. Sequence variations within the S gene directly determine viral antigenic features, host tropism, and the capacity to evade humoral immunity induced by commercial vaccines [6]. Proteolytic processing cleaves the S protein into S1 and S2 subunits. The extracellular S1 domain harbours the receptor-binding domain (RBD) as well as three well-characterized neutralizing epitopes (COE, SS2, SS6), and constitutes the major mutational hotspot across the viral genome [7,8].

Global PEDV strains are classified into genotypes G1 and G2 based on full-length S-gene sequence homology. The G2 clade can be further divided into four independent subclusters: G2a, G2b, G2c, and G2d [9,10,11]. Prior to 2010, the G1a prototype strain CV777 was predominant within Chinese pig herds. Following the nationwide spread of virulent variants, G2 strains gradually supplanted G1 strains as the dominant circulating population [12]. Recent multi-provincial surveillance data indicate that G2c is currently the most widely distributed subcluster in China, whereas novel G2d variants are frequently recovered from south-western provinces including Sichuan and Guangxi [13,14].

Homologous recombination serves as a key driver of genomic diversity among field PEDV populations [15,16]. The viral RNA-dependent RNA polymerase (RdRp) lacks proofreading activity, which results in frequent point mutations and template-switch-mediated recombination, particularly within the highly variable S gene [17]. Previous nationwide sequencing surveys reported that most G2c strains originate from recombination events between G2b and S-INDEL strains, while G2d variants arise from genetic exchange between G2a and S-INDEL isolates [18,19]. Regional monitoring work performed in Chongqing also identified obvious S-gene recombination signals, and a national dataset containing 347 samples confirmed broad circulation of chimeric G2c strains [20,21]. The majority of existing surveillance studies adopt cross-sectional sampling strategies; multi-year continuous investigations tracking viral adaptive changes within a single closed farm under fixed vaccination schemes remain scarce. This limits our understanding of how long-term immune selection reshapes strain composition and antigenic properties of circulating PEDV.

Vaccination remains the core preventive measure for mitigating PED outbreaks in intensive pig farms. Since 1994, inactivated and live-attenuated vaccines based on the G1a prototype strain CV777 have been commercially available in China. A bivalent vaccine targeting the G2b strain AJ1102 was subsequently released in late 2017 [22,23]. Although standardized sow immunization protocols have been widely implemented in large-scale farms, recurrent PED outbreaks still occur. This phenomenon can largely be attributed to progressive antigenic drift among circulating field isolates [24,25]. Persistent vaccine-derived immune pressure accelerates the accumulation of mutations and recombination within the S gene, generating variant viruses with reduced sensitivity to neutralization by vaccine-elicited polyclonal antibodies [26].

In the present study, full-length S-gene sequencing and bioinformatic analyses were performed for eight PEDV field sequences collected from one commercial pig farm in Guangxi during 2021–2025. This work describes the longitudinal genetic characteristics of these sequences, including phylogenetic classification, recombination signatures, and amino acid variations in neutralizing epitopes and N-glycosylation sites. The field-based data provide information for rational vaccine-strain evaluation and on-farm biosecurity management.

## 2. Materials and Methods

### 2.1. Sample Collection and Farm Vaccination Regimen

This study was conducted on a commercial pig farm housing 12,500 sows (approximately 312,500 weaned piglets annually, PSY 25). From 2021 to 2025, PEDV surveillance was performed on periparturient sows in farrowing rooms using anal swabs, yielding 29,120 samples. Annual testing and positive results were: 5809/755 (13.00%), 5796/994 (17.15%), 5913/917 (15.50%), 5863/879 (14.99%), and 5739/776 (13.52%) for 2021–2025, respectively, with an overall positive rate of 14.84%. This dataset represents farm-level epidemiological background.

In this study, the term “closed farm” refers to a commercial pig farm that operates a self-contained breeding herd with internal replacement gilts and does not introduce live pigs from external sources for production purposes. However, the farm may occasionally introduce external semen or replacement boars under strict quarantine and testing protocols. Despite stringent biosecurity measures, indirect introduction of pathogens via personnel, vehicles, or fomites cannot be completely excluded.

Piglet samples were collected during diarrhea outbreak investigations in farrowing units, not through fixed-interval surveillance; therefore, annual piglet positive rates are unavailable. All piglet samples were individual specimens from neonatal piglets ≤7 days old, including diarrheal feces from live piglets and small-intestinal tissues from naturally deceased piglets. No pooled sampling was used, and no animals were euthanized for research purposes. Affected piglets showed watery diarrhea and vomiting, with morbidity approaching 100% in affected litters and a case-fatality rate of 90–100%; these data apply only to outbreak units.

Eight representative samples were selected for full-length S-gene sequencing based on: (1) coverage of different calendar years; (2) Ct < 30; (3) absence of co-infection signals. No virus isolation was performed. The term “isolate” refers to field-sample-derived S-gene sequences.

Sows were routinely vaccinated against PRRSV, classical swine fever, pseudorabies, foot-and-mouth disease (O + A), Japanese encephalitis, and porcine parvovirus. PEDV prophylaxis consisted of live-attenuated vaccine AJ1102 at 30 days pre-partum and inactivated vaccine CV777 at 14 days pre-partum.

All specimens were residual clinical samples from routine surveillance. No experimental animals were used, and no animals were euthanized for this project. Nucleic-acid extraction was performed under BSL-2 conditions. Ethical approval was not required for residual diagnostic samples. S-gene sequences were deposited in GenBank under accession numbers PZ791501–PZ791508 (Submission ID: SUB16376704).

### 2.2. Full-Length S-Gene Segmented Amplification and Sanger Sequencing

Four overlapping primer pairs were used to amplify the complete S open reading frame (Table 1). Total viral RNA was extracted using TRIzol (Thermo Fisher Scientific, Waltham, MA, USA) following the single-step method of Chomczynski and Sacchi [27]. First-strand cDNA was synthesized using the HiScript II RT Kit (Vazyme Biotech, Nanjing, China).

PEDV positivity was confirmed by SYBR Green-based real-time PCR targeting the N gene, using primers described by Granda Moncayo et al. (2023) [28], with an annealing temperature optimized to 58 °C. Primer sequences were DEPNF397 (5′-AGATTGTTGAACCTAACACA-3′) and DEPNR565 (5′-ATTATTATTGCCTCCTCTGT-3′), amplifying a 169 bp fragment. Each 20 µL reaction contained 10 µL 2× SYBR Green master mix, 0.4 µM each primer, 2 µL cDNA, and nuclease-free water. Cycling conditions were 95 °C for 3 min; 40 cycles of 95 °C for 10 s, 58 °C for 30 s, and 72 °C for 30 s; final extension at 72 °C for 5 min. Melting-curve analysis confirmed specificity. Samples with Ct < 30 were selected for S-gene sequencing. Positive and negative controls were included in each run. qPCR was performed on an ABI 7500 instrument.(Applied Biosystems, Foster City, CA, USA).

Purified PCR amplicons were sequenced bidirectionally by Tsingke Biotechnology (Beijing, China) on an ABI 3730xl platform (Biosystems, Foster City, CA, USA). Raw reads were checked with Chromas (2.6.6.), and sequences were assembled in Lasergene (SeqMan v7.1). Primer regions were trimmed manually.

### 2.3. Phylogenetic Reconstruction and Pairwise Homology Calculation

Reference S-gene sequences representing canonical subclusters (G1a CV777, G1c OH851, G2b AJ1102, G2c NH-TA2020, G2d CH/HNBR/01/2021) were downloaded from GenBank (accession numbers in Table S1). Neighbour-joining phylogenetic trees were constructed in MEGA 11 with 1000 bootstrap replicates to evaluate branch reliability [29,30]. Pairwise nucleotide- and amino acid-identity matrices were generated using the MegAlign module within Lasergene v7.1 [31].

### 2.4. Homologous Recombination Detection

Recombination screening was performed in RDP4 v4.101 incorporating seven independent detection algorithms (RDP, GENECONV, BootScan, MaxChi, Chimaera, SiScan, and 3Seq) with default settings and a threshold *p*-value of 0.01 [32]. Only recombination events supported by at least five of the seven methods were considered credible. Putative parental sequences were GDS31 (G2b-like) and CH-GXLB-202112 (G2d-like), referred to as parental relatives rather than actual parents. Breakpoint estimates were approximately 880 nt for single-break recombinants and approximately 880 nt and 3850 nt for the dual-break recombinant CH-GXLB-202412. Similarity plots were produced with SimPlot++ v3.5.1 under the F84 substitution model; sliding-window size = 500 bp, step size = 100 bp. [33].

### 2.5. Full-S-Protein Multiple Alignment and Mutation Annotation

Complete S-protein amino acid sequences (residues 1–1386) of field isolates and reference strains were aligned in Jalview v2.11.3. All non-synonymous substitutions, insertions and deletions were annotated. Specialized analysis focused on the COE (499–638 aa), SS2 (748–755 aa), and SS6 (764–771 aa) epitope regions [7,8].

### 2.6. Solvent-Accessible Surface Area (SASA) Quantification

The published S-protein crystal structure of NH-TA2020 was used as the structural template. SASA values for wild-type and mutated antigen-related residues were calculated in PyMOL v2.5.

Calculation formula:

Relative SASA increase (%) = [(Mutant SASA − Wild-type SASA)/Wild-type SASA] × 100

### 2.7. In Silico Antigenic Index Prediction

The Jameson–Wolf algorithm implemented in the Protean module of Lasergene was applied to generate continuous antigenic-index profiles, so as to identify antigenic discrepancies between field isolates and vaccine strains [31,34].

### 2.8. N-Linked Glycosylation Site Prediction

N-glycosylation sequons were predicted using the NetNGlyc-1.0 online server (cut-off threshold = 0.5). Sites with jury score ≥ 9/9 or potential score > 0.75 were defined as high-confidence glycosylation sites. Patterns of glycosylation-site gain and loss were summarised to explore their potential roles in viral antigenic changes [35,36].

## 3. Results

### 3.1. Sequence Homology and Phylogenetic Classification of Farm Isolates

**Figure 1:**
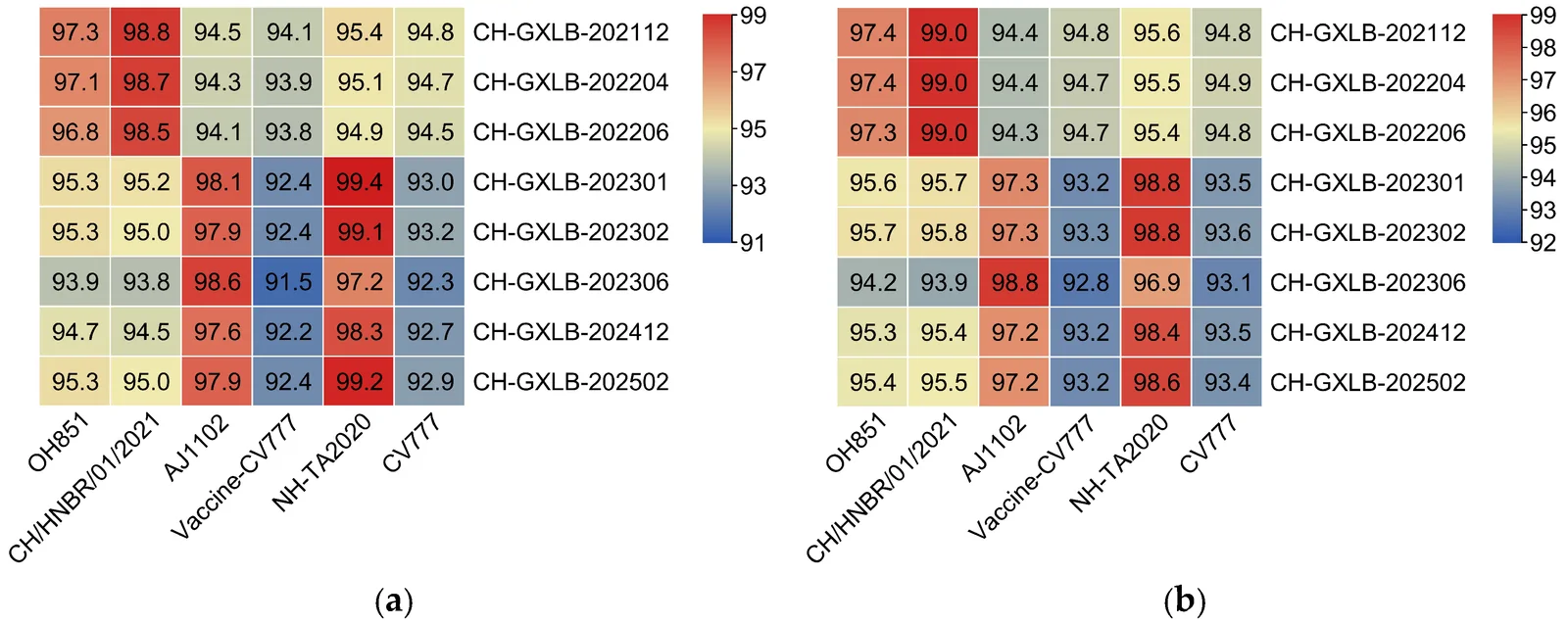
Nucleotide and amino acid identity analysis of the S gene between eight Guangxi PEDV strains and reference strains: (**a**) Nucleotide identities. (**b**) Amino acid identities. CV777 is highlighted in red. Values represent percentage identity calculated by MegAlign (Lasergene v7.1).

**Figure 2:**
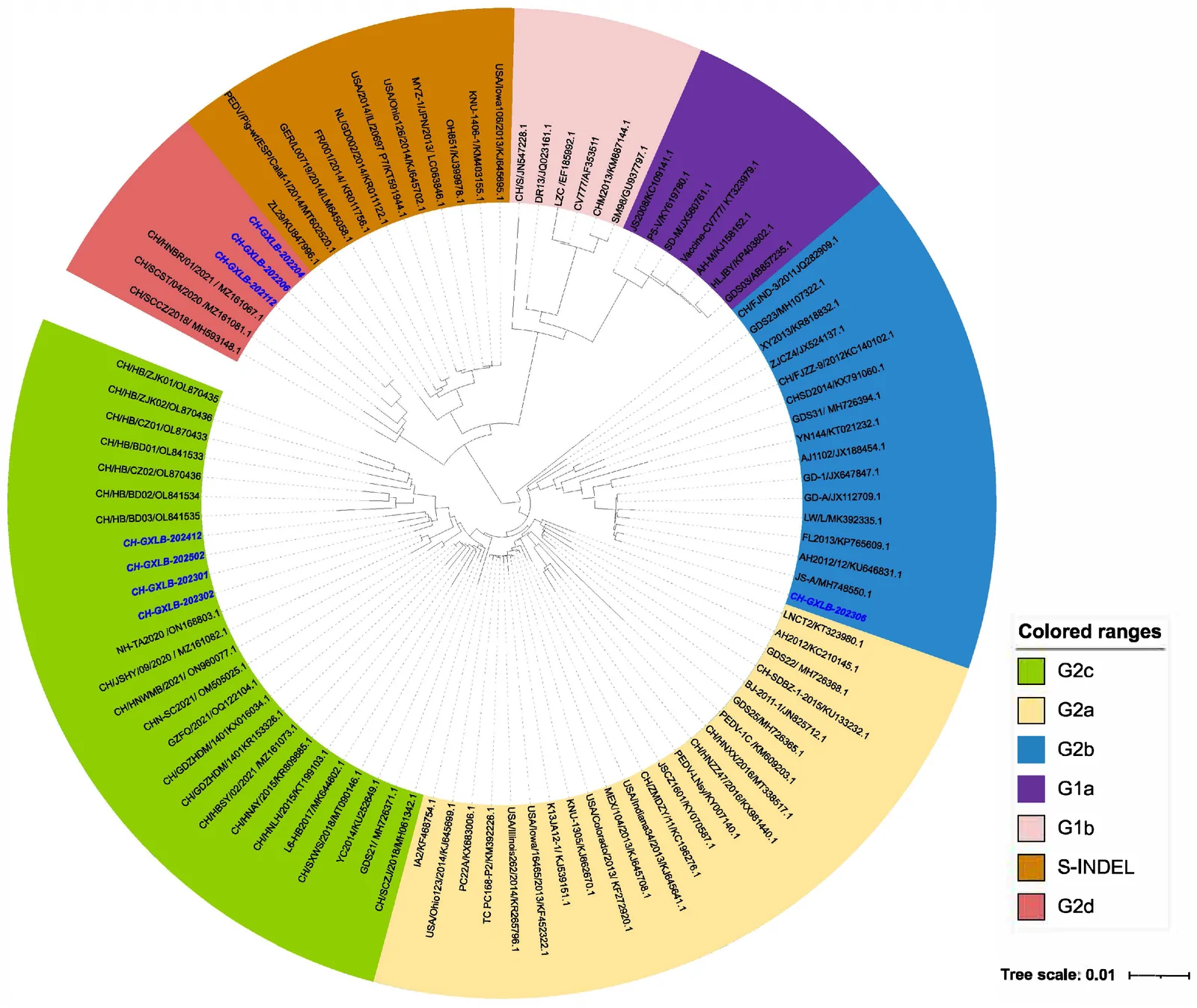
Phylogenetic tree of PEDV based on the S gene.

Pairwise identity comparison indicated that the eight Guangxi field sequences shared 92.8–99.0% nucleotide identity and 91.5–99.0% amino acid identity relative to global reference strains. Every sequence exhibited a maximum nucleotide homology of only 94.9% compared with the classical CV777 vaccine strain. Amino acid identity values were consistently lower than corresponding nucleotide-identity values, indicating the accumulation of non-synonymous mutations (Figure 1). Neighbour-Joining phylogenetic clustering assigned all eight sequences exclusively to the G2 genotype. They fell into three subclusters; no G1 sequences were detected from this farm (Figure 2): G2c subcluster (four sequences: CH-GXLB-202301, CH-GXLB-202302, CH-GXLB-202412, CH-GXLB-202502): closely clustered with the prototype G2c strain NH-TA2020, with pairwise nucleotide identity of 98.4–98.8%. These sequences were detected in 2023, 2024, and 2025. G2d subcluster (three sequences: CH-GXLB-202112, CH-GXLB-202204, CH-GXLB-202206) showed high genetic consistency with the G2d reference strain CH/HNBR/01/2021 (99.0% nucleotide identity). These sequences were detected in 2021 and 2022. The single G2b sequence (CH-GXLB-202306) displayed the highest sequence similarity to the vaccine backbone strain AJ1102 (98.8% nucleotide identity) and was detected in 2023. All farm-derived sequences formed monophyletic branches that were evolutionarily distant from the G1 CV777 vaccine clade.

### 3.2. Identification of Inter-Subtype Recombination Events in G2c Isolates

**Figure 3:**
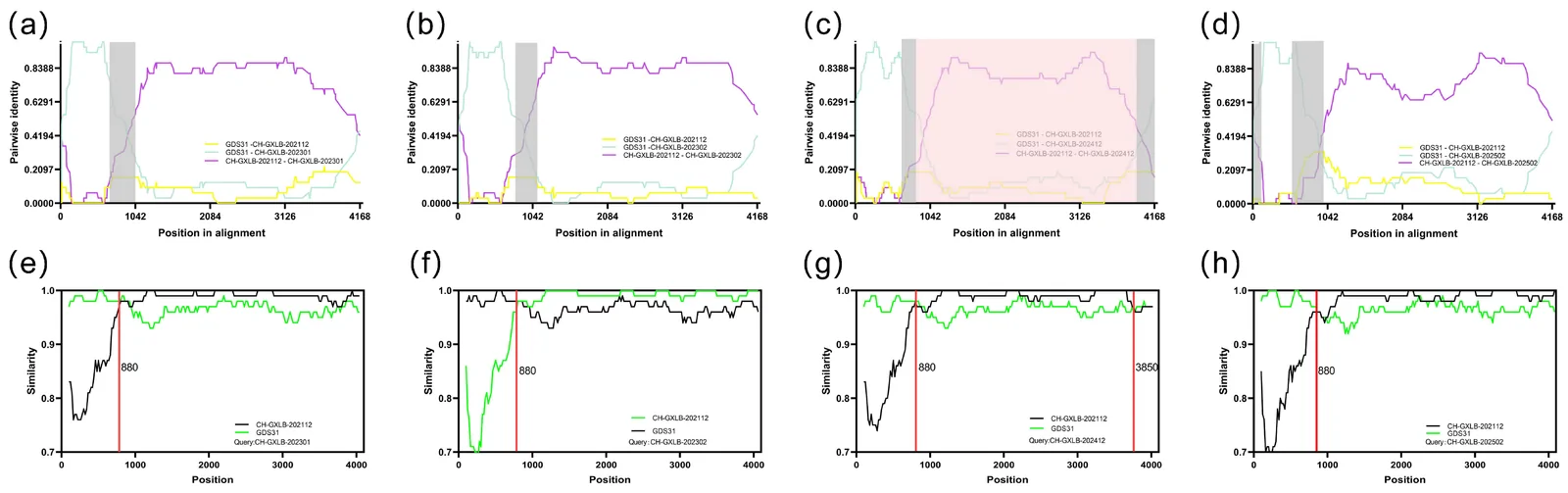
Recombination analysis based on the S gene. (**a**–**d**) SimPlot similarity plots for CH-GXLB-202301 (**a**), CH-GXLB-202302 (**b**), CH-GXLB-202412 (**c**), and CH-GXLB-202502 (**d**) compared with GDS31 and CH-GXLB-202112. The grey-shaded region marks the uncertain breakpoint zone (700–1042 nt). The pink-shaded region in panel (**c**) corresponds to the 880–3850 nt fragment inherited from a CH-GXLB-202112-like sequence. (**e**–**h**) Similarity plots of the S genes of CH-GXLB-202301 (**e**), CH-GXLB-202302 (**f**), CH-GXLB-202412 (**g**), and CH-GXLB-202502 (**h**) compared with GDS31 and CH-GXLB-202112. Vertical red lines indicate the estimated breakpoint positions (880 nt for single-break recombinants; 880 nt and 3850 nt for the dual-break recombinant CH-GXLB-202412).

Recombination screening was performed using seven algorithms (RDP, GENECONV, BootScan, MaxChi, Chimaera, SiScan, and 3Seq) implemented in RDP4 v4.101 with default settings and a threshold *p*-value of 0.01. Only recombination events supported by at least five of the seven methods were considered credible. All four G2c sequences were supported by at least five algorithms with *p* < 0.01. For CH-GXLB-202301 (single-break recombinant), the estimated breakpoint was approximately 880 nt (minimum *p* = 4.210 × 10^−26^). For CH-GXLB-202302 (single-break recombinant), the estimated breakpoint was approximately 880 nt (minimum *p* = 2.199 × 10^−25^). For CH-GXLB-202502 (single-break recombinant), the estimated breakpoint was approximately 880 nt (minimum *p* = 3.278 × 10^−26^). For CH-GXLB-202412 (dual-break recombinant), the estimated breakpoints were at approximately 880 nt and 3850 nt (minimum *p* = 1.314 × 10^−33^). The putative parental sequences were GDS31 (G2b-like) and CH-GXLB-202112 (G2d-like), referred to as parental relatives rather than actual parents. For CH-GXLB-202412, the regions 0–880 nt and 3850–4168 nt were inherited from a GDS31-like sequence, whereas the central fragment spanning 880–3850 nt was derived from a CH-GXLB-202112-like sequence. The 700–1042 nt interval represents the uncertain breakpoint zone and is shown as a grey-shaded region in Figure 3. These results reveal sequence-level recombination signatures within the G2c sequences. All four G2c sequences contained recombination breakpoints within the S1-NTD region, with G2b-like and G2d-like sequences as parental relatives.

### 3.3. Subcluster-Specific Amino Acid Mutations Within Core Neutralizing Epitopes

**Figure 4:**
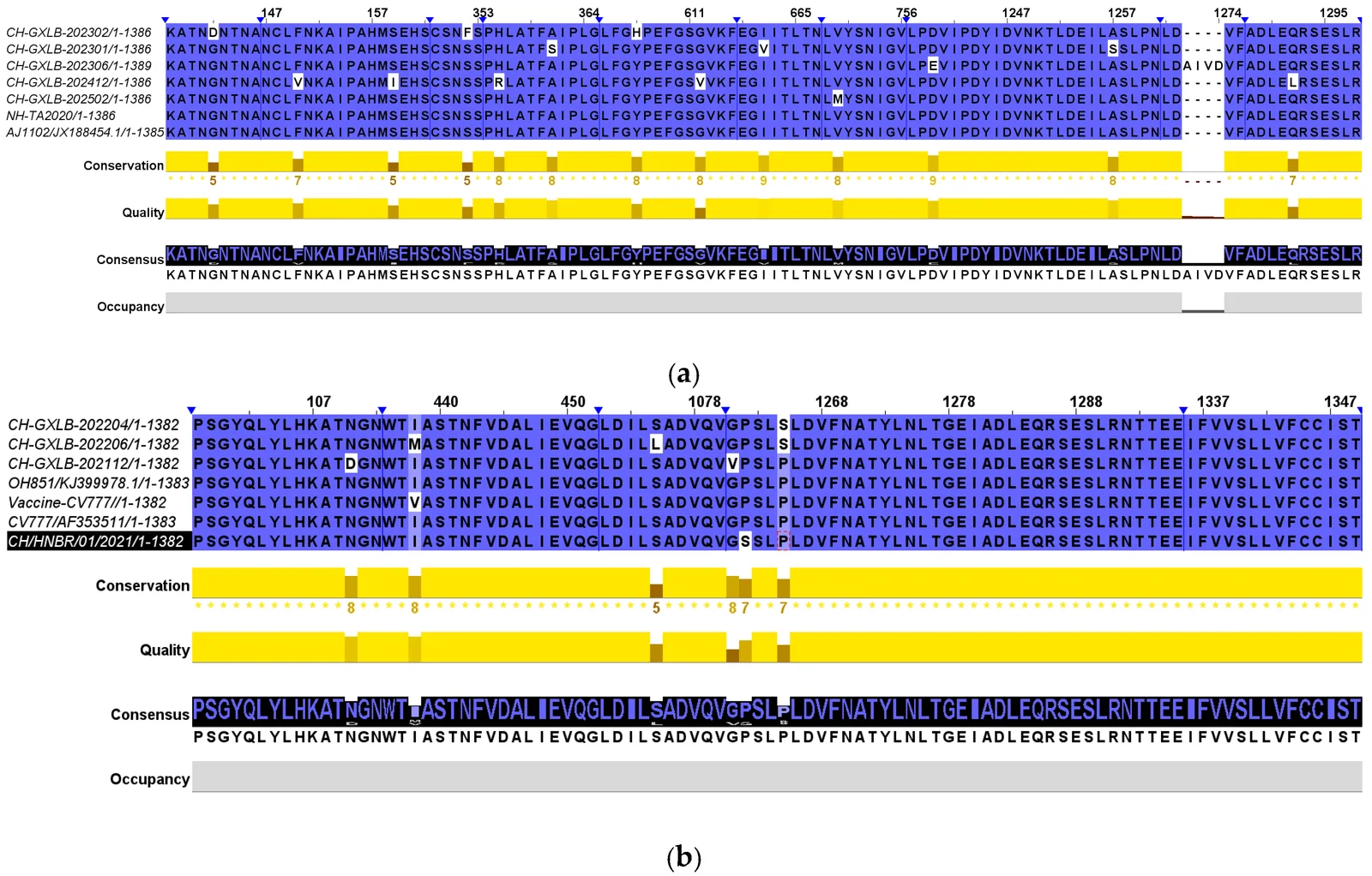
Alignment of the deduced amino acid sequences of the S gene. (**a**) Amino acid sequence alignment of G2d subcluster strains showing conserved residues. The 1271AIVD insertion in G2b is boxed in blue. (**b**) Amino acid sequence alignment between G2c and G2b subclusters with key epitope substitutions highlighted in red box (Y606H, G612V, V750M, P766R). Asterisks (*) indicate fully conserved residues across all aligned sequences.

Multiple-sequence alignment of full-length S-protein sequences for the present isolates and reference strains revealed divergent mutational landscapes among the three co-circulating subclusters (Figure 4). The G2b isolate CH-GXLB-202306 exhibited the highest mutation burden, including 10 scattered non-synonymous substitutions plus a unique four-residue insertion (1271AIVD1274) located within the HR2 fusion domain. The exclusive P766R substitution adjacent to the fusion-peptide region was detected only within this strain. All four G2c recombinants carried signature epitope substitutions absent in commercial vaccine strains CV777 and AJ1102: Y606H within the COE core in CH-GXLB-202302; G612V within the COE core in CH-GXLB-202412; and the rare V750M substitution within the SS2 linear epitope in CH-GXLB-202502. Strains belonging to the G2d subcluster displayed very high sequence conservation, harbouring only zero to two sporadic amino acid changes without fixed mutations within core neutralizing-epitope domains.

Published functional studies have demonstrated that single-residue substitutions within COE and SS2 domains can markedly reduce binding affinity for neutralizing monoclonal antibodies [7,8]. This observation strongly implies that these fixed substitutions may confer viral potential to evade humoral immunity induced by herd vaccination.

### 3.4. Structural, Antigenic and Glycosylation Alterations Triggered by Signature Mutations

To further evaluate the potential impacts of key mutations on S-protein function and antigenicity, complementary structural-prediction analyses were performed covering solvent accessibility, antigenic index, and N-glycosylation-site remodelling.

#### 3.4.1. Quantitative SASA Structural Changes

**Figure 5:**
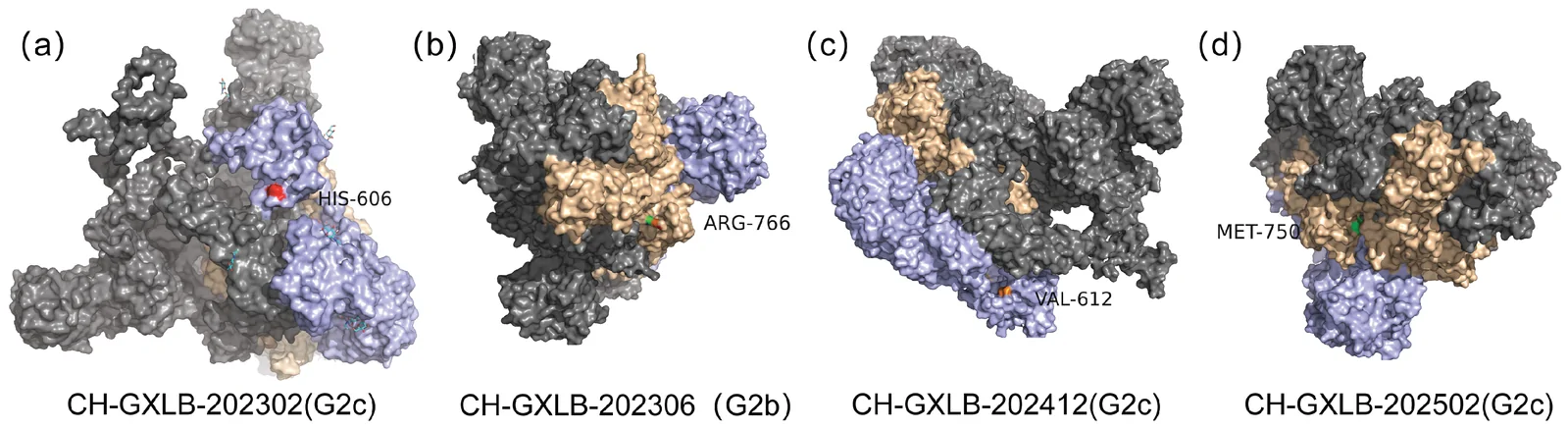
Three-dimensional structural comparison of the PEDV S protein. Homology modeling was performed using the S protein trimer structure of the G2c reference strain NH-TA2020 as the template in PyMOL. The S protein trimer is displayed in surface representation, with the three subunits shown in light gray and the A chain highlighted in green cartoon representation to facilitate visualization of the spatial positions of mutated residues. Mutated residues are shown as ball-and-stick models and colored according to subcluster origin: magenta for the G2b-specific mutation (P766R), and cyan/green for G2c recombinant mutations (Y606H, G612V, V750M). (**a**) CH-GXLB-202302 (G2c): spatial distribution of Y606H (COE region) and G612V (COE region) on the S1 subunit surface. (**b**) CH-GXLB-202306 (G2b): unique localization of P766R adjacent to the fusion peptide region on the S2 subunit. (**c**) CH-GXLB-202412 (G2c): spatial position of G612V (COE region). (**d**) CH-GXLB-202502 (G2c): localization of V750M within the SS2 epitope (S2 region). All mutated residues are located on the exposed surface of the S protein trimer, and the alterations in side-chain exposure may affect the spatial conformation of antibody recognition targets. Red dashed circles indicate the functional epitope regions (COE, SS2) containing the mutation sites. Quantitative SASA data are presented in Table 2.

SASA values were calculated in PyMOL for four key mutation sites located within or adjacent to neutralizing epitopes: Y606H, G612V, V750M, and P766R (Table 2, Figure 5). All four mutations produced increased SASA to varying degrees. The P766R substitution in CH-GXLB-202306 yielded a 300.5% relative increase (141.8 → 442.3 Å^2^), and G612V in CH-GXLB-202412 increased solvent accessibility by 184.9% (36.1 → 221.0 Å^2^). Three-dimensional structural modelling (Figure 5) confirmed that all mutated residues are positioned on the exposed outer surface of the S-protein trimer, within or adjacent to the COE, SS2, and SS6 neutralizing-epitope regions. These alterations in side-chain exposure may remodel the spatial conformation of antibody-recognition sites, potentially interfering with the binding efficiency of neutralizing antibodies and contributing to potential immune evasion phenotypes [7,8].

#### 3.4.2. Antigenic Index Disparity

**Figure 6:**
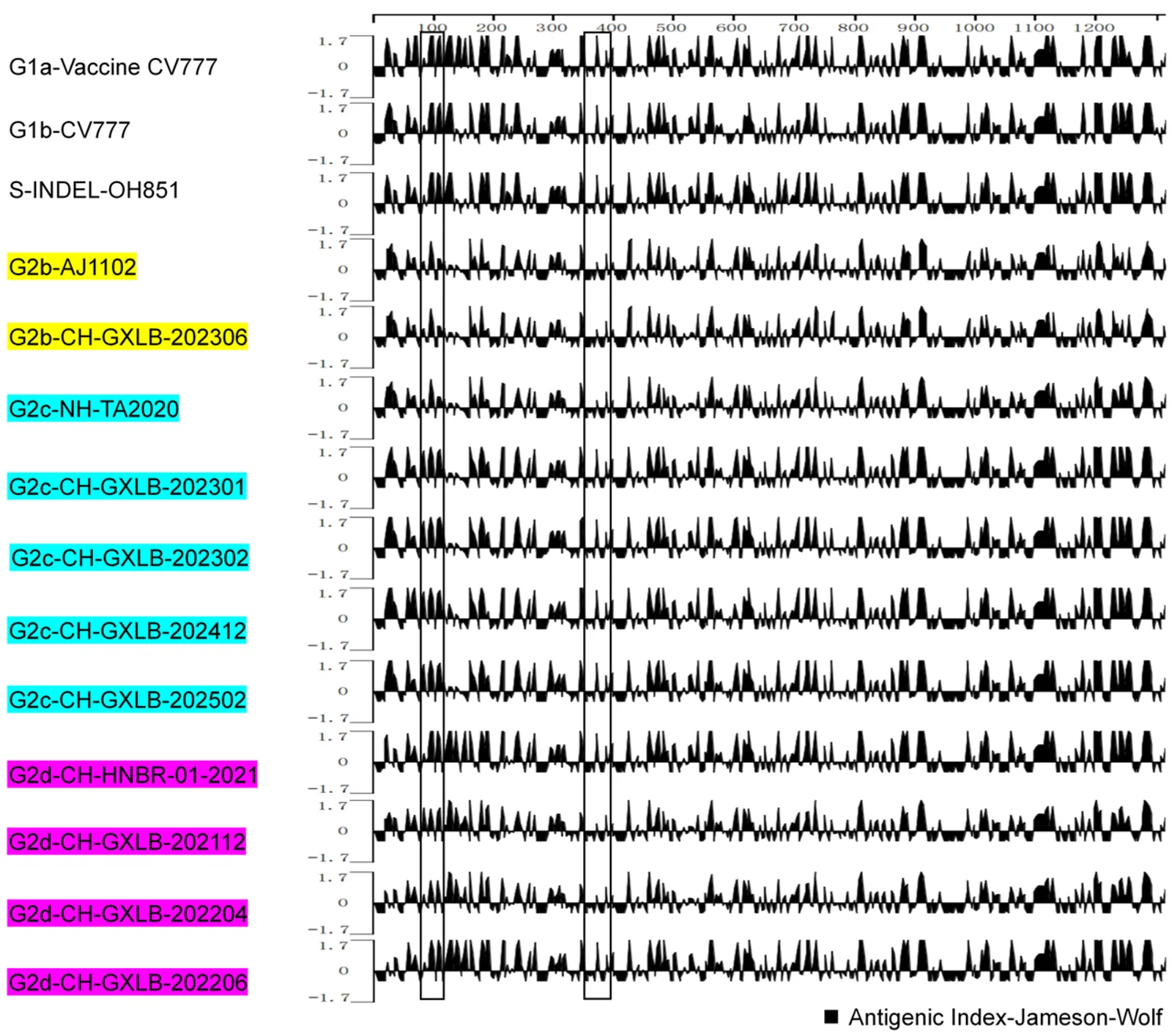
Differences in antigenic index of the S protein. Antigenic index profiles of S protein amino acid sequences. The antigenic index was calculated using the Jameson–Wolf algorithm implemented in the Protean module of DNASTAR Lasergene v7.1. Regions above the zero line indicate predicted antigenic sites. Rectangular boxes mark regions with antigenic differences among tested strains. Recombinant strains are plotted in blue and magenta; reference strains are plotted in black and yellow.

To verify whether these structural changes are accompanied by antigenic alterations, antigenic-index profiles for each strain were predicted using the Jameson–Wolf algorithm [31,34]. Results (Figure 6) demonstrated that recombinant G2c strains (CH-GXLB-202301, -202302, -202412) exhibited pronounced antigenic divergence compared with the reference strain NH-TA2020 within the 300–400 aa region of the S1 subunit. This region overlaps with strain-specific amino acid substitutions S352F and A361S. Meanwhile, the endemic G2d isolate CH-GXLB-202112 presented minor antigenic shifts in the 80–120 aa region relative to other G2d strains and the reference strain CH/HNBR/01/2021. These observations suggest that recombination-introduced mutations may reshape the local antigenic topography of the spike glycoprotein.

#### 3.4.3. Conserved N-Glycosylation Drift Signature

NetNGlyc-based prediction identified subcluster-specific N-glycosylation profiles across PEDV isolates (Table 3). A conserved glycan shift distinguished G2b/G2c from G2d/G1 lineages: G2b and G2c isolates consistently lost N57 and N127 while gaining N62 and N118, whereas G2d and G1 strains retained the ancestral pattern. Within G2c, additional microevolutionary variations were observed: CH-GXLB-202502 uniquely lost N300 and gained N787; CH-GXLB-202302 accumulated substitutions at residues 300 (I → M), 351 (N → F), 1009 (I → Y), and 1232 (L → Y); and CH-GXLB-202412 uniquely lost the highly conserved N1294 site. Within G2d, the three Guangxi isolates uniformly displayed a substitution at residue 425 (V → G) compared with the reference strain, suggesting a shared local adaptive change. These gain/loss events may alter protein folding and glycan shielding of adjacent neutralizing epitopes, potentially facilitating viral antigenic adaptation under immune pressure [35,36].

## 4. Discussion

### 4.1. Longitudinal Monitoring Reveals Temporal Changes in PEDV Subcluster Detection

This five-year sampling provides field data on PEDV genetic characteristics in a single commercial swine farm under a stable sow vaccination scheme. Phylogenetic analysis detected three G2 subclusters at different time points: G2d sequences in 2021–2022, G2c sequences in 2023–2025, and a single G2b sequence in 2023. Because sampling was outbreak-triggered and not continuous, these observations should be interpreted as detection trends rather than evidence of competitive replacement or dominance.

Similar shifts in PEDV subcluster detection have been reported in national surveillance [37]. Large-scale S-gene sequence analysis and multi-provincial surveillance data indicate that GIIc/G2c is among the most frequently detected PEDV subtypes in China, accounting for 26.06% of GII subtypes and 40% of circulating strains [38]. However, most cross-sectional surveys cannot capture within-farm viral population dynamics. Our data indicate that different PEDV subclusters can co-circulate or appear sequentially in a vaccinated herd, but the factors driving these changes remain unclear.

The detection of a G2b-like sequence in 2023 indicates that a virus related to the vaccine backbone AJ1102 was present on the farm. Previous studies have reported that exotic viruses can be introduced into pig farms via replacement gilts, vehicles, or feed [2,12]. However, the present data cannot determine the source of this virus or whether it was introduced from outside the farm. Co-circulation of different subclusters may provide opportunities for recombination, but the present data cannot establish that co-infection, recombination, or genotype replacement occurred on this farm.

We hypothesize that vaccination-associated immune pressure may influence the selection of circulating variants. Experimental studies have shown that under prolonged antibody pressure, the S protein 1273 site is prone to mutation (A1273P), which significantly enhances the virus’s ability to escape polyclonal antibody neutralization in vitro and in vivo while maintaining virulence [39]. However, whether the variants observed in this study have similar functional properties still requires further field evidence and functional validation.

### 4.2. Recombination Signatures in G2c Sequences Suggest G2b- and G2d-Related Parental Relatives

Homologous recombination is an important source of genetic diversity in coronaviruses. Previous studies have commonly attributed the origin of G2c to recombination between G2b and S-INDEL strains [13,18,40]. In contrast, our data identified sequence-level recombination signatures in G2c sequences, suggesting G2b-like and G2d-like sequences as parental relatives, with breakpoints located within the S1-NTD region (700–1042 nt). Both single-break and dual-break recombinant architectures were observed.

These results suggest that G2c may have multiple recombination origins. However, sequence-based recombination detection can only identify putative recombination signatures; it cannot determine the timing or location of the recombination events. The reference sequences GDS31 and CH-GXLB-202112 are parental relatives, not the actual viruses involved in recombination. Co-circulation of different subclusters in the same farm may provide opportunities for recombination, but the present data cannot establish that recombination occurred on this farm.

These findings suggest that the recombination origins of G2c may be more diverse than previously recognized. They also highlight the importance of continued S-gene molecular surveillance in vaccinated herds for early detection of novel recombinant variants. Further field sampling and functional experiments are needed to determine whether these recombination signatures are associated with antigenic changes or immune evasion.

### 4.3. Core-Epitope Mutations Reshape Spike Antigen Topography

The COE and SS2 domains are key linear neutralizing epitopes of the PEDV S-protein; single-residue changes within these domains can reduce monoclonal-antibody binding [6,36]. The G2c-specific substitutions Y606H, G612V (COE) and V750M (SS2) are absent in vaccine backbones CV777 and AJ1102.

SASA analysis provides structural clues for potential antigenic adaptation. Glycine-612 has a minimal side chain; the G612V substitution introduces bulky hydrophobic residues and distorts local three-dimensional epitope geometry, potentially disrupting antibody–antigen complementarity. Although the V750M mutation produced only moderate SASA elevation (+10.1%), the SS2 epitope spans merely eight residues; even single-site alteration may impair antibody-recognition efficiency. The G2b-unique P766R caused the largest observed SASA increase (+300.5%) and localizes adjacent to the fusion-peptide region, which may modulate viral membrane-fusion efficiency alongside antigenic properties, reflecting divergent evolutionary trajectories between G2b and G2c variants coexisting on this farm.

### 4.4. N-Glycosylation Remodelling Cooperates with Epitope Mutation to Drive Dual Antigenic-Adaptation Potential

N-linked glycosylation forms a steric glycan shield across coronavirus spike surfaces to conceal antibody-targeted epitopes and may mediate humoral-antigenic adaptation [35]. Compared with G2d and G1 strains, which retained glycosylation at N57 and N127 and lacked N62 and N118, G2b and G2c isolates consistently lost N57/N127 while gaining N62/N118, indicating a lineage-associated glycan shift distinguishing the G2b/G2c clade, a pattern consistent with glycan-shift profiles reported in Guangxi PEDV surveillance [41]. Within the G2c subcluster, additional microevolutionary variations were observed: CH-GXLB-202502 uniquely acquired N787 while losing N300; CH-GXLB-202302 accumulated substitutions at residues 300, 351, 1009, and 1232; and CH-GXLB-202412 uniquely lost N1294. The three Guangxi G2d isolates consistently displayed a substitution at N425 (V → G), suggesting a shared local adaptive change. Combined epitope mutations and glycosylation drift may thus constitute a dual-layer molecular strategy supporting sustained circulation of recombinant PEDV under high-level herd-vaccination pressure.

### 4.5. Practical Production and Vaccine-Development Implications

Pairwise sequence comparison confirmed that all farm sequences share ≤94.9% nucleotide identity relative to the classical CV777 vaccine strain. This finding reflects genetic differences between contemporary field variants and traditional G1-based vaccine platforms. Although the AJ1102 (G2b) vaccine shows high sequence similarity to the single G2b sequence recovered during 2023, epitope remodelling and glycosylation variation may limit its predicted cross-protective efficacy against G2c and G2d sequences [42,43]. These genetic differences highlight the importance of continued surveillance. Vaccine-development programmes should consider G2c and G2d strains as potential vaccine backbones, but functional validation is required.

Currently, no commercial vaccine specifically targeting G2c strains is available on the market. The G2c candidate strain NH-TA2020 has been shown to induce lactogenic immunity and provide partial protection against homologous and G2d challenges in experimental studies [40]. For farms experiencing recurrent PED outbreaks despite vaccination with G1a- or G2b-based vaccines, a rational approach would be to first evaluate the genetic match between circulating strains and available vaccine strains. In practice, when a significant antigenic mismatch is confirmed, some farms have adopted autogenous vaccines or controlled feedback protocols under strict biosecurity as emergency measures. However, these approaches currently lack standardized protocols, and their safety and efficacy require further field validation. The development of G2c-based vaccine candidates should be prioritized for future vaccine updates [44,45].

### 4.6. Limitations of This Study

There are several notable limitations in the current study. First, this work represents a retrospective molecular epidemiological investigation based on field clinical samples. All conclusions regarding antigenic alteration and immune evasion capacity are derived from sequence analysis and structural bioinformatics prediction only; follow-up in vitro neutralization experiments together with animal challenge models are necessary to validate these hypothesized characteristics. Second, our surveillance was performed at a single commercial farm. The evolutionary pattern described here may not fully represent PEDV epidemic scenarios across broader geographical regions. Third, only samples from diseased piglets were collected; we lack virological and serological data from asymptomatic carrier pigs within the herd, which restricts our comprehensive understanding of the complete transmission chain of PEDV circulation. Fourth, because piglet sampling was outbreak-triggered, the number of piglet samples varied among years and cannot be used to estimate annual piglet-level prevalence. Even with these constraints, our longitudinal field data still offer valuable real-world insights into PEDV genetic dynamics under practical farming conditions.

## 5. Conclusions

In summary, our five-year field surveillance described the genetic characteristics of PEDV sequences within a large-scale commercial pig farm in Guangxi. G2d sequences were detected in 2021–2022, and G2c sequences were detected in 2023–2025, with a single G2b sequence detected in 2023. Recombination signatures were identified in G2c sequences, with G2b- and G2d-related parental relatives. Several amino acid substitutions were located within or adjacent to neutralizing epitopes, and N-glycosylation site variations were observed. Bioinformatic predictions suggest that these changes may alter antigenic properties, but functional validation is still required. This study highlights the importance of continuous PEDV surveillance at pig farms and provides field-derived data for vaccine-strain evaluation and on-farm disease control.

## Figures and Tables

**Table 1:** Oligonucleotide primers for full-length PEDV S gene amplification.

Name	Sequence (5′-3′)	Product Length/bp
PEDV-SF	GTACTTTCAACACTTAGCCTACCA	310
PEDV-SR	GTTGTAGGTAGGTGTATAAACATA	
PEDV-S1F	ATGAAGTCTTTAACTTACTTCTGG	1150
PEDV-S1R	CGTAGATGGTATACTTGGTGCAC	
PEDV-S2F	CTGTGTTGACACTAGACAATTTA	1100
PEDV-S2R	TACTCAGTGACTGTTAAAGGTGG	
PEDV-S3F	GCCATTTCTAGTTCTATTGATGA	1080
PEDV-S3R	TTCTTTCGGTTTTCTAGGTTCAA	
PEDV-S4F	GTGGCAGATCTAGTCTGTGCAC	1050
PEDV-S4R	TCACTGCACGTGGACCTTTTCAA	

**Table 2:** Solvent-accessible surface area (SASA) before and after mutation calculated via PyMOL (quantitative data).

Strains	Mutation	NH-TA2020 SASA (Å^2^)	Mut SASA (Å^2^)	Relative Change (%)
CH-GXLB-202302	Y606H	186.721	254.314	+67.593
CH-GXLB-202412	G612V	36.051	220.982	+184.931
CH-GXLB-202306	P766R	141.82	442.337	+300.517
CH-GXLB-202502	V750M	152.241	162.296	+10.055

Solvent-accessible surface area (SASA, Å^2^) and relative change rate of residues at critical antigenic sites of the wild-type and mutant S protein. Relative change rate (%) = (Mutant SASA − Wild-type SASA)/Wild-type SASA × 100%.

**Table 3:** High-confidence N-glycosylation site variations in G1, G2b, G2c, and G2d PEDV strains predicted by NetNGlyc 1.0.

Strains	N-Glycosylation Site Variations																			
	57	62	112	118	127	216	230	300	324	344	351	381	425	787	873	1009	1196	1231	1260	1294
AJ1102 (G2b)	/	NSTW	/	NATA	/	NVTS	/	NQTI	NDTS	NFSF	NSSD	NSTV	NFTG	/	NFTN	NITS	/	NLTR	NRTG	NTTE
																				1298
CH-GXLB-202306 (G2b)	/	NSTW	/	NATA	/	NVTS	/	NQTI	NDTS	NFSF	NSSE	NSTV	NFTG	/	NFTN	NITS	/	NLTR	NRTG	NTTE
																		1232	1261	1295
NH-TA2020 (G2c)	/	NSTW	/	NATA	/	NVTS	/	NQTI	NDTS	NLSF	NSSD	NSTV	NFTG	/	NFTN	NITS	NHTA	NLTR	NRTG	NTTE
CH-GXLB-202301 (G2c)	/	NSTW	/	NATA	/	NVTS	/	NQTI	NDTS	NLSF	NSSD	NSTV	NFTG	/	NFTN	NITA	NHTA	NLTR	NRTG	NTTE
CH-GXLB-202502 (G2c)	/	NSTW	/	NATA	/	NVTS	/	/	NDTS	NLSF	NSSD	NSTV	NFTG	NFSV	NFTN	NITS	NHTA	NLTR	NRTG	NTTE
CH-GXLB-202412 (G2c)	/	NSTW	/	NATA	/	NVTS	/	NQTI	NDTS	NLSF	NSSD	NSTV	NFTG	/	NFTN	NITS	NHTA	NLTR	NRTG	/
CH-GXLB-202302 (G2c)	/	NSTW	/	NATA	/	NVTS	/	NQTM	NDTS	NLSF	NFSD	NSTV	NFTG	/	NFTN	NITS	NYTA	NLTR	NRTG	NTTE
						212	229	296	320	340	347	377	421		869	1005	1192	1228	1257	1291
CH/HNBR/01/2021 (G2d)	NSSS	/	/	/	NKTL	NVTS	/	NQTI	NDTS	NLSF	NSSN	NSTV	NFTV	/	NFTN	NITS	NHTA	NLTR	NRTG	NTTE
CH-GXLB-202204 (G2d)	NSSS	/	/	/	NKTL	NVTS	/	NQTI	NDTS	NLSF	NSSN	NSTV	NFTG	/	NFTN	NITS	NHTA	NLTR	NRTG	NTTE
CH-GXLB-202206 (G2d)	NSSS	/	/	/	NKTL	NVTS	/	NQTI	NDTS	NLSF	NSSN	NSTV	NFTG	/	NFTN	NITS	NHTA	NLTR	NRTG	NTTE
CH-GXLB-202112 (G2d)	NSSS	/	/	/	NKTL	NVTS	/	NQTI	NDTS	NLSF	NSSN	NSTV	NFTG	/	NFTN	NITS	NHTA	NLTR	NRTV	NTTE
Vaccine-CV777 (G1a)	NSSS	/	NTSA	/	NKLT	NVTS	NCSG	NQTM	NDTF	NLSF	NSSD	/	NFTG	/	NFTN	NITS	/	NLTS	/	NTTE
						213		297	321	341	348	378	422		870	1006	1193	1229	1258	1292
OH851 (S-INDEL)	NSSS	/	/	/	NKLT	NVTS	NCSG	NQTM	NDTS	NLSF	NSSD	NSTV	NFTG	/	NFIT	NITS	NHTA	NLTR	NRTG	NTTE
CV777 (G1b)	NSSS	/	/	/	NKTL	NVTS	NCTG	NHTM	NDTS	NLSF	NSSD	NSTV	NFTG	/	NFTN	NITS	/	NLTS	NRTG	NTTE

“/” indicates the absence of the corresponding glycosylation site in the strain. Red font indicates variations in glycosylation site motifs compared with the reference strains (including both loss of glycosylation sites and amino acid substitutions within the N-X-S/T sequon). Positions are numbered according to the full-length S protein (1–1386 aa). Amino acid sequence motifs are presented in the single-letter code (N-X-S/T, where X is any amino acid except proline). For G2b and G2c strains, the upper row of position numbers applies; for G2d, G1a, S-INDEL, and G1b strains, the lower row applies.

## Data Availability

The genome sequences of the eight PEDV isolates characterized in this study have been deposited in the NCBI GenBank database under accession numbers PZ791501–PZ791508 (Submission ID: SUB16376704). The datasets generated and/or analyzed during the current study are available from the corresponding author upon reasonable request.

## Supplementary Materials

The following supporting information can be downloaded at: https://www.mdpi.com/article/10.3390/ani16203262/s1, Table S1: Reference PEDV S-gene sequences used for phylogenetic and recombination analyses.

## Abbreviations

The following abbreviations are used in this manuscript:

PEDV	Porcine epidemic diarrhea virus
S	Spike
COE	Core neutralizing epitope
SASA	Solvent-accessible surface area
NTD	N-terminal domain
RdRp	RNA-dependent RNA polymerase
