# Supplementary material for: Longitudinal Genotype Replacement, Recombination and Neutralizing Epitope Variations in Porcine Epidemic Diarrhea Virus at a Commercial Pig Farm in Guangxi, China
Source: Animals (Basel). 2026 Oct 16;16(20):3262. doi: 10.3390/ani16203262 (PMC13700771; doi:10.3390/ani16203262)
Supplement: Supplementary file 1 [file animals-16-03262-s001.zip › animals-4573454-supplementary.pdf]

Table S1. Reference PEDV S-gene sequences used for phylogenetic and recombination analyses.

No.	Strain	GenBank accession no.
1	JSCZ1601	KY070587.1
2	NH-TA2020	ON168803.1
3	K13JA12-1	KJ539151.1
4	USA/Colorado/2013	KF272920.1
5	USA/Iowa/16465/2013	KF452322.1
6	IA2	KF468754.1
7	GDS22	MH726368.1
8	GDS25	MH726365.1
9	USA/Iowa106/2013	KJ645695.1
10	MYZ-1/JPN/2013	LC063846.1
11	GER/L00719/2014	LM645058.1
12	NL/GD002/2014	KR011122.1
13	FR/001/2014	KR011756.1
14	AH-M	KJ158152.1
15	Vaccine-CV777	KT323979.1
16	SD-M	JX560761.1
17	P5-V	KY619780.1
18	SM98	GU937797.1
19	CHM2013	KM887144.1
20	CV777	AF353511.1
21	DR13	JQ023161.1
22	JS2008	KC109141.1
23	GD-A	JX112709.1
24	GDS23	MH107322.1
25	CHSD2014	KX791060.1
26	CH/SCCZ/2018	MH593148.1
27	CH/SCZJ/2018	MH061342.1
28	CH/SCST/04/2020	MZ161081.1
29	GDS21	MH726371.1
30	JS-A	MH748550.1
31	GDS31	MH726394.1
32	OH851	KJ399978.1
33	AH2012	KC210145.1
34	AH2012/12	KU646831.1
35	AJ1102	JX188454.1
36	BJ-2011-1	JN825712.1
37	CH/HNAY/2015	KR809885.1
38	CH/HNBR/01/2021	MZ161067.1
39	CH/HNLH/2015	KT199103.1
40	CH/HNWMB/2021	ON960077.1
41	CH/JSHY/09/2020	MZ161082.1
42	CHN-SC2021	OM505025.1
43	CH/HB/CZ01	OL870433.1
44	CH/HB/CZ02	OL870436.1
45	CH/HB/ZJK01	OL870435.1
46	CH/HB/ZJK02	OL870436.1
47	CH/HB/BD01	OL841533.1
48	CH/HB/BD02	OL841534.1

49	CH/HB/BD03	OL841535.1
50	LZC	EF185992.1
51	ZL29	KU847996.1
52	KNU-1406-1	KM403155.1
53	CH-SDBZ-1-2015	KU133232.1
54	LNCT2	KT323980.1
55	PC22A	KX683006.1
56	CH/ZMDZY/11	KC196276.1
57	PEDV-LNSy	KY007140.1
58	CH/HNZZ47/2016	KX981440.1
59	L6-HB2017	MK644602.1
60	YC2014	KU252649.1
61	CH/HBSY/02/2021	MZ161073.1
62	CH/FJZZ-9/2012	KC140102.1
63	CH/GDZHDM/1401	KR153326.1
64	TC_PC168-P2	KM392226.1
65	MEX/104/2013	KJ645708.1
66	XY2013	KR818832.1
67	ZJCZ4	JX524137.1
68	USA/Ohio126/2014	KJ645702.1
69	USA/Illinois262/2014	KR265796.1
70	PEDV-1C	KM609203.1
71	GDS03	AB857235.1
72	HLJBY	KP403802.1
73	CH/HNXX/2016	MT338517.1
74	CH/SXWS/2018	MT090146.1
75	USA/2014/IL	KT591944.1
76	USA/Indiana34/2013	KJ645641.1
77	USA/Ohio123/2014	KJ645699.1
78	PEDV/Pig-wt/ESP/Calaf-1/2014	MT602520.1
79	FL2013	KP765609.1
80	YN144	KT021232.1
81	GZFQ/2021	OQ122104.1
82	KNU-1305	KJ662670.1
83	LW/L	MK392335.1
84	GD-1	JX647847.1
85	CH/FJND-3/2011	JQ282909.1
86	CH/S	JN547228.1
